# Low dose hCG supplementation in a Gn-RH-agonist trigger protocol is associated with worse pregnancy outcomes: a retrospective cohort study

**DOI:** 10.1186/s40738-021-00104-8

**Published:** 2021-05-28

**Authors:** Maren Shapiro, Phillip Romanski, Ann Thomas, Andrea Lanes, Elena Yanushpolsky

**Affiliations:** 1 Obstetrics & Gynecology, Brigham and Women’s Hospital and Harvard Medical School, Boston, MA USA; 2 Center for Reproductive Health, University of California, 499 Illinois Street, 6th floor, San Francisco, CA 94158 USA; 3 Ronald O. Perelman and Claudia Cohen Center for Reproductive Medicine, Weill Cornell Medical College, New York, NY USA

**Keywords:** GnRH agonist, IVF, OHSS, Dual trigger, Luteal phase support

## Abstract

**Background:**

A number of studies have looked at dual triggers with hCG and GnRH agonist (GnRHa) in varying doses, but the question remains: what is the optimal dose of hCG to minimize ovarian hyperstimulation syndrome (OHSS) and still offer adequate pregnancy rates? The purpose of this study was to compare pregnancy and OHSS rates following dual trigger for oocyte maturation with GnRHa and a low-dose hCG versus hCG alone. A secondary objective was the assess pregnancy outcomes in subsequent frozen cycles for the same population.

**Methods:**

A total of 963 women < 41 years old, with a BMI 18–40 kg/m^2^ and an AMH > 2 ng/mL who underwent fresh autologous in vitro fertilization (IVF) with GnRH antagonist protocol at a University-based fertility center were included in this retrospective cohort study. Those who received a low dose dual trigger with hCG (1000u) and GnRHa (2 mg) were compared to those who received hCG alone (10,000u hCG/250-500 μg Ovidrel). Differences in implantation rates, pregnancy, live birth, and OHSS were investigated.

**Results:**

The dual trigger group was younger (mean 33.6 vs 34.1 years), had a higher AMH (6.3 vs 4.9 ng/mL,) more oocytes retrieved (18.1 vs 14.9) and a higher fertilized oocyte rate (80% vs 77%) compared with the hCG only group. Yet, the dual trigger group had a lower probability of clinical pregnancy (gestational sac, 43.4% vs 52.8%) and live birth (33.4% vs 45.8%), all of which were statistically significant. There were 3 cases of OHSS, all in the hCG-only trigger group. In subsequent frozen cycles, pregnancy rates were comparable between the two groups.

**Conclusions:**

The dual trigger group had a better prognosis based on age and AMH levels and had better stimulation outcomes, but significantly worse pregnancy outcomes, suggesting the low dose hCG (1000u) in the dual trigger may not have provided adequate luteal support, compared to an hCG-only trigger (10,000u hCG/250-500 μg Ovidrel). Interestingly, the pregnancy rates were comparable in subsequent frozen cycles, further supporting the hypothesis that the issue lies in inadequate luteal phase support, rather than embryo quality. Based on these findings, our program has changed the protocol to 1500u of hCG in a dual trigger.

## Introduction

In ovarian stimulation cycles for assisted reproductive technology (ART), there is a delicate balance between achieving adequate stimulation for sufficient oocyte yield and hyperstimulation. Ovarian hyperstimulation syndrome (OHSS) is a side effect of the exogenous human chorionic gonadotropin (hCG) hormones used to trigger oocyte maturation [1]. Although only 0.1 to 0.2% of all in vitro fertilization (IVF) cycles are associated with severe OHSS, the consequences can be devastating, including renal failure, hypovolemic shock, thromboembolic events, acute respiratory distress syndrome and rarely even death [2–4]. Because of the gravity of such consequences in an otherwise generally healthy patient population, there has been a push in recent years by members of the ART community towards “OHSS Free Clinics” [5, 6].

As the exogenous hCG trigger appears to be the major contributor to OHSS, presumably due to its longer half-life, there has been an effort to develop stimulation protocols that avoid its use, the most effective of which appears to be a GnRH antagonist cycle with a GnRH agonist (GnRHa) trigger for final follicle maturation [7–10]. Initial studies found that with a GnRHa trigger, the risk for OHSS was essentially eliminated, due to a combination of the short half-life of pituitary LH compared with hCG and pituitary desensitization from the agonist leading to rapid luteolysis [11, 12]. Unfortunately, triggering with GnRHa alone was associated with significantly lower pregnancy and live birth rates compared with hCG trigger in fresh embryo transfer cycles [9]. The hypothesis is that the rapid luteolysis does not allow for adequate estrogen and progesterone levels in the luteal phase to support the endometrium and promote embryo implantation [13, 14]. A number of different protocols have been proposed to provide this additional luteal phase support, including progesterone supplementation [15], hCG *after* trigger [16], and more recently, a dual trigger with both hCG and GnRHa [17–21].

A number of studies have looked at dual triggers in varying doses with various outcome measures, but the question remains--what is the optimal regimen [17–21] Specifically, what is the lowest effective dose of hCG which offers comparable pregnancy rates while minimizing OHSS risk? At our practice, we use a low dose of hCG [1000u] as a dual trigger with GnRHa and see virtually no OHSS. To date, only a handful of studies have looked at a low-dose hCG/GnRHa dual trigger, but in the majority of them the primary outcome was oocyte yield rather than pregnancy rate, a much more clinically significant outcome [22–25].

The objective of this present study was to assess stimulation, pregnancy, and OHSS outcomes following dual trigger with a low-dose hCG protocol compared with an hCG only trigger in GnRH antagonist fresh embryo transfer cycles. A secondary objective was the assess pregnancy outcomes in subsequent frozen cycles for the same patient population, with an ultimate goal of determining whether 1000u of hCG in a dual trigger provides adequate luteal phase support to allow for appropriate live birth rates for this patient population.

## Methods

### Study design

A retrospective cohort study was performed using our prospectively maintained departmental infertility database, with supplemental information abstracted from our hospital electronic medical records, at the Center for Infertility and Reproductive Surgery at Brigham and Women’s Hospital in Boston, Massachusetts. All women who underwent stimulation and oocyte retrieval for the purposes of autologous fresh IVF and IVF/intracytoplasmic sperm injection (ICSI) cycles performed between 1/1/2012 and 5/31/2017 were evaluated. This study was approved by the Partners Human Research Committee at the Brigham and Women’s Hospital (Protocol #2017P001579).

### Participants

The study included all patients < 41 years old who underwent IVF or IVF/ICSI cycles using a GnRH antagonist protocol and were triggered with either hCG alone (hCG trigger group: 10,000u hCG/250-500 μg Ovidrel) or both a low dose of hCG and a GnRHa (dual trigger group: 1000u hCG + 2 mg GnRHa). In the hCG trigger group, Ovidrel dose was varied based on BMI—those with a BMI < 30 received 250u and those with a BMI ≥ 30 received 500u. Only the initial fresh autologous cycles were included in the primary analysis, while the first subsequent frozen cycle was included in the secondary analysis. Women were excluded if they were felt to be very poor responders, based on the following criteria: severe overweight or underweight (BMI < 18 or > 40 kg/m^2^), AMH < 2.0. Those with uterine factor infertility as a primary infertility diagnosis were excluded, as were freeze all cycles.

### Clinical protocols

Stimulation protocols were restricted to gonadotropin-releasing hormone (GnRH) antagonist protocols. Following retrieval, oocytes were either inseminated in groups (3–5 oocytes) or underwent intracytoplasmic sperm injection (ICSI). A fertilization check was then performed at 16–18 h and zygotes with 2 pronuclei (2pn) were cultured individually in 25 ul droplets of global total medium (Life Global Group, Cooper Surgical; Guilford, CT) overlain with mineral oil in benchtop incubators maintained in dry atmosphere consisting of 5% O_2_ and 6–7% CO_2_, balanced with N_2_. Embryos were moved to fresh global total medium on day 3. Per internal protocol, the number of 2pn zygotes and age were used to determine the number of embryos transferred and if a patient received a day 3 versus day 5 transfer, with those having fewer than 6 2pn zygotes receiving a day 3 transfer.

Multiple embryo quality parameters were used to determine embryo quality. Embryo quality on day 3 was determined by cell number, fragmentation score and symmetry of blastomeres. All embryos that continued to day 5 were evaluated and scored for development (arrested, early or late morula or blastocyst with extent of expansion) and quality of the inner cell mass, and the trophectoderm, based on previously described protocols [26]. The best quality embryos available were always chosen for transfer, in both hCG and dual trigger cycles for both day 3 and day 5 transfer groups. There were no changes to the culture system or laboratory standard operating procedures during the study time period.

The trigger type used was decided by a patient’s primary physician based on personal risk assessment for OHSS, with age < 35, AMH > 3.4, estradiol on day of trigger > 3500, a diagnosis of PCOS with BMI < 25 considered to be risk factors for OHSS [27, 28]. Those felt to be at higher risk for OHSS were typically assigned a GnRH-a trigger with a 1000u hCG co-trigger while those felt to be at low risk for OHSS received an hCG only trigger. Luteal support was provided with vaginal progesterone gel (one applicator daily), beginning 2 days after oocyte retrieval and continued up to 10 weeks of gestation for the hCG group as per the standard protocol in our program. The dual trigger group received enhanced luteal phase support in the form of oral estradiol 3 mg twice daily in addition to the daily vaginal progesterone supplementation, beginning 1 day after oocyte retrieval. Estrogen supplementation was discontinued once there was a positive pregnancy test.

Given overwhelming evidence in the literature that intramuscular progesterone and vaginal progesterone are equally efficacious forms of luteal support in stimulated IVF.cycles [29–32] and that there is no need for additional luteal phase estrogen supplementation in cycles that use an hCG only trigger [32], this has become the standard practice in our clinic.

### Outcome variables

The primary outcome measured was live birth rate. Secondary outcomes included implantation rate, clinical pregnancy, ongoing pregnancy, and OHSS. Live birth rate was defined as delivery of a live child after 24 weeks gestation. Implantation rate was calculated by dividing the number of gestational sacs by the number of embryos transferred. Pregnancy was defined as the presence of a positive hCG. Clinical pregnancy was defined as the presence of a gestational sac. Ongoing pregnancy was defined as those who graduated to Obstetric care, which in our practice is typically at around 8 weeks gestational age. For the outcome of OHSS, all patients who were at high risk (day of trigger estradiol > 3500 or those who received cabergoline as a post-trigger medication) were identified. Their electronic medical charts were reviewed in detail for a formal diagnosis of OHSS. Diagnosis and severity of OHSS was determined by the primary provider based on ASRM guidelines [28].

### Statistical analyses

Frequencies and proportions were reported for categorical variables. Means and standard deviations were reported for continuous variables. Log-binomial and Poisson regression models were used to estimate the relative risks or counts (RR), with a 95% confidence interval (CI). For rate ratios the log of the appropriate denominator term was included in Poisson models as an offset variable. Patient age, ovarian diagnosis (the presence of diminished ovarian reserve or ovarian dysfunction), and AMH were included in all models a priori to calculate the adjusted relative risks, counts, or rate ratios (aRR). Race, BMI, Day 3 FSH, use of ICSI, day of transfer, endometrial thickness, and number of embryos transferred were tested in the models as covariates and included only if found to be significant confounders. In the analysis of pregnancy outcome variables, the number of embryos transferred was also included in the adjusted models. Statistical analyses were performed using SAS software version 9.4,© 2013 SAS Institute Inc.

## Results

### Demographics and stimulation parameters

A total of 525 hCG trigger cycles and 438 dual trigger cycles met inclusion criteria for the initial analysis. Demographics and baseline characteristics are presented in Table 1. Women in the dual trigger group were younger than those in the hCG trigger group (mean 33.6 vs 34.1 years). The dual trigger group had a higher average AMH (6.3 vs 4.9 ng/mL). The two groups were comparable with regards to prior pregnancy history and race/ethnicity.

**Table 1:** Demographic and baseline characteristics of women undergoing IVF between 1/1/2012 and 5/31/2017.

	Trigger Type	
	hCG trigger (*N* = 525) n (%)	Dual trigger (*N* = 438) n (%)
Woman’s Age (years)		
< 30	95 (18.1%)	99 (22.6%)
31–34	211 (40.2%)	176 (40.2%)
35–37	132 (25.1%)	124 (28.3%)
38–39	53 (10.1%)	30 (6.9%)
40–40.9	34 (6.5%)	9 (2.1%)
Race/Ethnicity		
Caucasian	356 (67.8%)	311 (71.0%)
African American	24 (4.6%)	16 (3.7%)
Hispanic	34 (6.5%)	13 (3.0%)
Asian	92 (17.5%)	77 (17.6%)
Other/declined	19 (3.6%)	21 (4.8%)
BMI (kg/m^2^)^a^	25.6 (5.0)	24.6 (4.4)
Type of infertility^b^		
Male factor	170 (32.4%)	145 (33.0%)
Tubal factor	53 (10.1%)	38 (8.7%)
Ovulation dysfunction	98 (18.7%)	99 (22.6%)
Endometriosis	38 (7.2%)	31 (7.1%)
Other	40 (7.6%)	43 (9.9%)
Unexplained	185 (35.2%)	152 (34.6%)
Nulligravida	303 (57.7%)	279 (63.7%)
Nulliparous	427 (81.3%)	364 (83.1%)
AMH^a^	4.9 (3.8)	6.3 (4.5)

^a^Presented as mean (standard deviation).

^b^Diagnoses are not mutually exclusive.

Stimulation parameters are presented in Table 2. Total days of stimulation and duration of GnRH antagonist treatment were comparable between the two groups as were estradiol levels, progesterone levels, and endometrial thickness on day of trigger. A greater proportion of the dual trigger group had a day 5 embryo transfer (74.7% vs 58.1%) compared to the hCG trigger group.

**Table 2:** Cycle characteristics of women undergoing IVF cycles.

	Trigger Type	
	hCG Trigger Mean (SD)	Dual trigger Mean (SD)
Total days on gonadotropins	11.9 (2.3)	11.6 (2.0)
E2 on trigger day (pg/mL)	1962 (787)	1996 (894)
Duration of GnRH-antagonist treatment (days)	4.7 (2.1)	4.5 (1.3)
Endometrial thickness on trigger day (mm)	11.1 (2.9)	10.7 (2.7)
Day of embryo transfer^a^		
Day 3	220 (41.9%)	111 (25.3%)
Day 5	305 (58.1%)	328 (74.7%)

^a^Presented as n (%).

### IVF outcomes

**Fig. 1:**
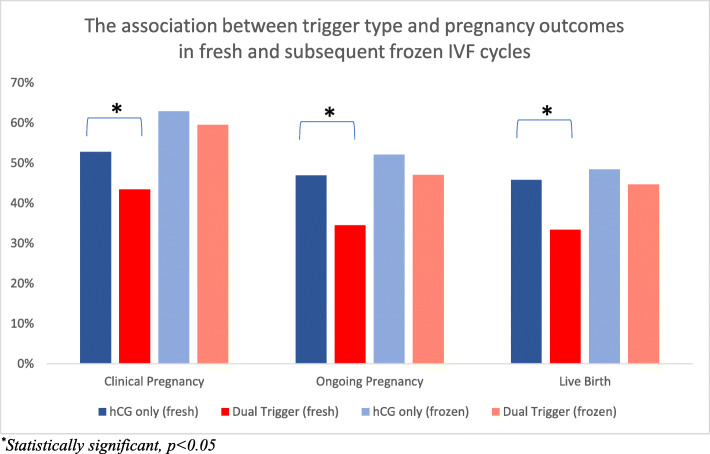
Compared to the hCG only trigger group, the dual trigger group had significantly lower clinical pregnancy, ongoing pregnancy and live birth rates infresh transfer cycles, but comparable rates in subsequent frozen cycles

Tables 3 and 4 and Fig. 1 show IVF outcomes in the two groups. The dual trigger group had more oocytes retrieved (18.1 vs 14.9, aRR 1.22, 95% CI 1.15–1.30) overall, which included more mature oocytes (13.5 vs 11.5) and a higher fertilized oocyte rate (0.80 vs 0.77, aRR 1.07, 95% CI (1.03–1.10). The dual trigger group had an average of 1.2 embryos transferred compared with 1.5 embryos for the hCG trigger group (aRR 0.85, 95% CI 0.81–0.89). Of note, all analyses were adjusted a priori for number of embryos transferred and age. Despite these better initial outcomes, the dual trigger group had a significantly lower probability of clinical pregnancy (43.4% vs 52.8%, aRR 0.80, 95% CI 0.70–0.91), ongoing pregnancy (34.5% vs 46.9%, aRR 0.72, 95% CI 0.61–0.85), and live birth (33.4% vs 45.8%, aRR 0.73, 95% CI 0.62–0.86). There were 3 cases of mild OHSS, all in the hCG trigger group.

**Table 3:** The association between trigger type and IVF outcomes.

	Trigger Type		
	hCG trigger (ref) Mean (SD)	Dual trigger Mean (SD)	Adjusted RR^**a**^ (95% CI)
Oocytes retrieved	14.9 (7.2)	18.1 (8.1)	1.22 (1.15, 1.30)
Mature oocytes	11.5 (6.1)	13.5 (6.6)	
Mature oocytes rate^b^	0.78 (0.18)	0.75 (0.18)	0.98 (0.94, 1.01)
Normally fertilized embryos (2PN)	8.7 (5.2)	11.0 (6.1)	
Fertilization rate^c^	0.77 (0.23)	0.80 (0.20)	1.07 (1.03, 1.10)
Blastocysts^d^	7.9 (4.0) *n = 305*	9.2 (4.7) *n = 328*	
Blastocyst conversion rate^e^	0.72 (0.21)	0.72 (0.20)	0.99 (0.95, 1.04)
OHSS	3 (0.6%)	0 (0%)	
Number of embryos transferred	1.5 (0.6)	1.2 (0.5)	0.85 (0.81, 0.89)

^a^Model adjusted for maternal age at retrieval, ovarian diagnosis and AMH. Tested covariates that did not show a significant association between the exposure and the outcome and thus were not included in the final model were race, BMI, day 3 FSH, use of ICSI, day of embryo transfer, endometrial thickness and number of embryos transferred.

^b^Mature oocyte rate defined as Mature oocytes/oocytes retrieved.

^c^Fertilization rate defined as 2PNs/oocytes retrieved.

^d^Only those with a day 5 transfer were included in the analysis.

^e^Blastocyst conversion rate defined as blastocysts/2PNs.

**Table 4:** The association between trigger type and pregnancy outcomes in fresh autologous embryo transfer cycles.

	Trigger Type		
	hCG trigger (ref) (***N*** = 525) n (%)	Dual trigger (***N*** = 438) n (%)	Adjusted RR^**a**^ (95% CI)
Pregnancy	328 (62.5%)	264 (60.3%)	0.95 (0.86, 1.06)
Clinical pregnancy	277 (52.8%)	190 (43.4%)	0.80 (0.70, 0.91)
Ongoing pregnancy	246 (46.9%)	151 (34.5%)	0.72 (0.61, 0.85)
Live birth	240 (45.8%)	146 (33.4%)	0.71 (0.60, 0.84)

^a^Model adjusted for maternal age at retrieval, ovarian diagnosis, AMH, and number of embryos transferred.

### Frozen cycles

A secondary analysis was performed comparing pregnancy outcomes between the two trigger groups in their first subsequent frozen cycle, with similar endometrial preparation protocols. These findings are shown in Table 5. There were 213 patients in the HCG trigger group and 217 patients in the dual trigger group who went on to have a frozen embryo transfers. In this cohort, the mean number of embryos transferred in the hCG trigger group was 1.33 (standard deviation 0.51) compared with 1.21 (standard deviation 0.44) in the dual trigger group. Unlike the fresh cycles, in the frozen cycles, pregnancy outcomes were similar in the two groups across all parameters, including clinical pregnancy (59.5% vs 62.9% aRR 0.94, 95% CI 0.80–1.09), and live birth (44.7%% vs 48.4%, aRR 0.91, 95% CI 0.73–1.11).

**Table 5:** The association between trigger type and pregnancy outcomes in the first subsequent frozen cycle.

	Trigger Type		
	hCG trigger *N* = 213 (ref) n (%)	Dual trigger *N* = 217 n (%)	Adjusted RR^**a**^
Pregnancy	150 (70.4%)	148 (68.2%)	0.97 (0.86, 1.11)
Clinical pregnancy	134 (62.9%)	129 (59.5%)	0.95 (0.81, 1.10)
Ongoing pregnancy	111 (52.1%)	102 (47.0%)	0.90 (0.75, 1.10)
Live birth	103 (48.4%)	97 (44.7%)	0.92 (0.75, 1.11)

^a^Model adjusted for maternal age at retrieval, ovarian diagnosis, and number of embryos transferred.

## Discussion

The primary aim of this study was to determine whether a low dose hCG dual trigger for ovulation maturation would provide adequate luteal phase support to sustain a successful pregnancy without increasing OHSS rates. We found that although women in the dual trigger group had a better prognosis based on age and AMH level and better stimulation outcomes, pregnancy outcomes were significantly lower across the board in comparison with the hCG only control group. The fact that this difference was not seen in subsequent frozen cycles further supports the hypothesis that 1000u hCG dose is not enough to provide adequate luteal phase support.

A GnRHa trigger is often used in IVF to reduce morbidity because it is associated with virtually no risk for OHSS [11]. This works very well in donor egg or freeze-all cycles, with comparable pregnancy rates to standard hCG-only triggers [33]. However, in fresh cycles, there are lower pregnancy rates and higher miscarriage rates [34, 35], likely due to inadequate luteal phase support [36]. Many strategies have been suggested to remedy this issue [12], including triggering with both GnRHa and a small dose of hCG, but optimal dose remains a question. Previous studies have shown that the addition of 1500u of hCG provides adequate luteal support to allow for comparable, if not higher, clinical pregnancy rates compared with hCG alone [37].

While other studies have looked at low-dose dual triggers with 1000u of hCG, to our knowledge, this is the largest study to date. Griffen et-al [24] compared live birth rates between those who received a dual trigger with low-dose hCG (1000u) versus GnRHa alone and found that the dual-trigger group had a significantly higher live birth rate with only one case of mild OHSS. Their study only looked at high responders who were at risk for OHSS and had a relatively small sample size (*n* = 68 for GnRHa alone and *n* = 34 for dual). Similarly, Shapiro et al. [22] in a correspondence in *Fertility and Sterility* also found that live birth rates were higher in those who received dual-trigger with low-dose hCG, except they varied the hCG dose (1000 to 2500 IU) based on the patient’s age and OHSS risk and had one patient with late-onset severe OHSS. Again, their comparator group was GnRH-a alone.

In addition to the larger sample size, one of the major differences in this study was that we used hCG only trigger as a control group, rather than GnRHa alone. As discussed above and supported by our cohort, GnRHa triggers are often used in women who are expected to be very high responders in order to ameliorate OHSS risk. Many of the traits that put one at risk for OHSS—such as young age, normal BMI, and high AMH—also make the GnRHa trigger group inherently a better prognosis group overall. Unsurprisingly, in our cohort, those in the dual trigger group were more likely to have a day 5 transfer than those in the hCG only trigger group. As better prognosis patients with more day 5 embryo transfers, those who received a dual trigger should have been expected to have had equivalent or better pregnancy outcomes than patients who received an hCG only trigger. While comparing pregnancy outcomes to a GnRHa trigger alone demonstrates that any additional luteal phase support, such as with an additional low-dose hCG trigger, will improve pregnancy outcomes, what is not known is whether the dose we use in our practice (1000u) is sufficient to offer the pregnancy rates that this good prognosis group should be achieving. In this study, we demonstrated that although the dual trigger group had a better prognosis, they had lower pregnancy rates than those receiving an hCG only trigger. This suggests that the 1000u hCG supplementation may not be providing adequate luteal support for optimal IVF cycle outcomes. Our practice found this evidence so compelling, that after the completion of this analysis, we have changed our dose of hCG to 1500u for dual trigger cycles.

With increasing doses of hCG also comes increasing risk for OHSS, so it’s important to weigh the benefit of improved stimulation outcomes with these risks. In our cohort, we did not find any clinically significant cases of OHSS in the dual trigger group, but we recognize that has not been the case for other cohorts**.** In a retrospective cohort study, O’neill et al. [25] found that the incidence of early OHSS was significantly higher after dual trigger with 1000u hCG than with GnRHa only trigger (8.6 vs 0%), although the absolute numbers were still low. They used weekly phone calls from nurses to assess for OHSS symptoms, so likely were able to capture more cases, but whether these were all clinically significant is unclear. Further, larger, studies are needed to fully assess this risk.

Strengths of this study include the large sample size, detailed outcome information, and the comparison of subsequent frozen cycles, which to our knowledge has not been documented before. Limitations include the retrospective design—namely that the type of trigger used was based on physicians’ assessment of OHSS risk and was not randomized. Moreover, because embryo quality was assessed using multiple parameters, we were not able to adjust for embryo quality in our modeling. Our study was conducted at one clinical practice, so results may not be generalizable to programs with differing IVF protocols. Finally, given OHSS is a rare outcome, the study was not powered to find a difference, but it is reassuring that there were no cases of clinically significant OHSS found in the dual trigger group.

## Conclusions

In conclusion, although dual trigger for oocyte maturation using a fixed low dose of hCG (1000u) as an adjuvant to GnRHa does not appear to increase the risk of clinically significant OHSS, it does not provide adequate luteal phase support, resulting in lower than expected pregnancy rates. Larger, prospective randomized controlled studies are needed to establish the optimal dose of hCG to both support early pregnancy development and offer an acceptably low risk for OHSS.

## Data Availability

The datasets generated and/or analyzed during the current study are not publicly available due to privacy concerns but are available from the corresponding author on reasonable request. Presented as a poster presentation at the American Society for Reproductive Medicine Scientific Congress and Expo in October 2018.

## Abbreviations

AMH: Antimullerian hormone
ART: Assisted reproductive technology
GnRHa: Gonadotropin releasing hormone-agonist
hCG: Human chorionic gonadotropin
IVF: In vitro fertilization
ICSI: Intracytoplasmic sperm injection
OHSS: Ovarian hyperstimulation syndrome
2pn: 2 Pronuclei
